# Protocol for Translabial 3D-Ultrasonography for diagnosing levator defects (TRUDIL): a multicentre cohort study for estimating the diagnostic accuracy of translabial 3D-ultrasonography of the pelvic floor as compared to MR imaging

**DOI:** 10.1186/1472-6874-11-23

**Published:** 2011-06-03

**Authors:** Kim JB Notten, Mirjam Weemhoff, Kirsten B Kluivers, Karlijn J Schweitzer, Femke Mulder, Jaap Stoker, Regina GH Beets-Tan, Jurgen J Futterer, Roy FA Vliegen, Johannes LH Evers, Gerold Link, Martin GM Bergmans, Paul HNM Kampschöer, Ed TCM Gondrie, Iris van Gestel, Ivo van Dooren, Carmen Dirksen, Luc JM Smits, Patrick M Bossuyt, Jan Paul WR Roovers

**Affiliations:** 1 Department of Obstetrics and Gynecology, Maastricht University Medical Centre, The Netherlands; 2 Department of Obstetrics and Gynecology, Radboud University Nijmegen Medical Centre, The Netherlands; 3 Department of Obstetrics and Gynecology, University Medical Centre Utrecht, The Netherlands; 4 Department of Obstetrics and Gynecology, Academic Medical Centre Amsterdam, The Netherlands; 5 Department of Radiology, Academic Medical Centre Amsterdam, The Netherlands; 6 Department of Radiology, Maastricht University Medical Centre, The Netherlands; 7 Department of Radiology, Radboud University Nijmegen Medical Centre, The Netherlands; 8 Department of Radiology, Atrium Medical Centre Parkstad Heerlen, The Netherlands; 9 Department of Epidemiology, Maastricht University Medical Centre, The Netherlands; 10 Department of Epidemiology, Academic Medical Centre Amsterdam, The Netherlands; 11 Department of Obstetrics and Gynecology, Laurentius Hospital Roermond, The Netherlands; 12 Department of Obstetrics and Gynecology, Atrium Medical Centre Parkstad Heerlen, The Netherlands; 13 Department of Obstetrics and Gynecology, Orbis Medical Centre Sittard, The Netherlands; 14 Department of Obstetrics and Gynecology, Viecuri Hospital Venlo, The Netherlands; 15 Department of Obstetrics and Gynecology, Sint Jans Gasthuis Hospital Weert, The Netherlands

## Abstract

**Background:**

Pelvic organ prolapse (POP) is a condition affecting more than half of the women above age 40. The estimated lifetime risk of needing surgical management for POP is 11%.

In patients undergoing POP surgery of the anterior vaginal wall, the re-operation rate is 30%. The recurrence risk is especially high in women with a levator ani defect. Such defect is present if there is a partially or completely detachment of the levator ani from the inferior ramus of the symphysis. Detecting levator ani defects is relevant for counseling, and probably also for treatment. Levator ani defects can be imaged with MRI and also with Translabial 3D ultrasonography of the pelvic floor.

The primary aim of this study is to assess the diagnostic accuracy of translabial 3D ultrasonography for diagnosing levator defects in women with POP with Magnetic Resonance Imaging as the reference standard. Secondary goals of this study include quantification of the inter-observer agreement about levator ani defects and determining the association between levator defects and recurrent POP after anterior repair. In addition, the cost-effectiveness of adding translabial ultrasonography to the diagnostic work-up in patients with POP will be estimated in a decision analytic model.

**Methods/Design:**

A multicentre cohort study will be performed in nine Dutch hospitals. 140 consecutive women with a POPQ stage 2 or more anterior vaginal wall prolapse, who are indicated for anterior colporapphy will be included. Patients undergoing additional prolapse procedures will also be included.

Prior to surgery, patients will undergo MR imaging and translabial 3D ultrasound examination of the pelvic floor. Patients will be asked to complete validated disease specific quality of life questionnaires before surgery and at six and twelve months after surgery. Pelvic examination will be performed at the same time points.

Assuming a sensitivity and specificity of 90% of 3D ultrasound for diagnosing levator defects in a population of 120 women with POP, with a prior probability of levator ani defects of 40%, we will be able to estimate predictive values with good accuracy (i.e. confidence limits of at most 10% below or above the point estimates of positive and negative predictive values).

Anticipating 3% unclassifiable diagnostic images because of technical reasons, and a further safety margin of 10% we plan to recruit 140 patients.

**Trial registration:**

Nederlands trial register NTR2220.

## Background

Pelvic organ prolapse (POP) is a condition affecting more than half of the women above age 40 [1]. The estimated lifetime risk of needing surgical management for pelvic organ prolapse is 11% [2].

In the Netherlands yearly 13,000 patients undergo surgical correction for POP [3]. These operations are known to have a re-operation rate of up to 30% because of primary failure or secondary recurrence of signs and symptoms of POP [2]. Because of this high re-operation rate, prolapse recurrence after pelvic floor surgery constitutes a major health care problem. Therefore identifying patients with an individual higher risk for recurrence of POP after surgery can possibly lead to individualized counseling and choice of treatment and possibly to reduction of the proportion of re-operations.

Several risk factors associated with surgical failure have been investigated in a number of studies.

Cystocele is the most commonly affected compartment in pelvic organ prolapse and is also the most prone for recurrence after surgery [4,5]. Younger women and women with a more advanced prolapse, a previous hysterectomy, and vaginal childbirth are at an increase risk of prolapse recurrence after surgery [6-9].

Recently, using Magnetic Resonance Imaging (MRI), trauma to the levator ani muscle has been shown to be a common consequence of vaginal delivery [10-12], affecting 20-30% of parous women as compared to 0% in nulliparous women [13]. Trauma to the levator ani muscle generally seems to manifest as a partial or complete detachment of the levator ani from the inferior ramus of the symphysis. [14]. A relationship has been established between levator defects and POP. This at least partly explains the link between vaginal childbirth and POP, and possibly also with POP recurrence after surgery [15,16].

In a population of urogynecological patients, women with levator defects postpartum were about twice as likely to show POP stage II or higher - especially cystocele and uterine prolapse - as those with an intact levator muscle, [15]. In a general population, women with POP appeared to have more levator defects than controls without POP (55% compared to 16%) [17].

In a previous study we showed anatomical recurrence of cystocele was associated with major levator defects with an odds ratio of 2.5 (95% confidence interval (CI) 1.1-5.7, p = 0.03) [18]. Dietz et al reported an even stronger association (OR 7.0, 95% CI 2.6, 19.1, p < 0.01) [19].

Recent advancements in imaging allow assessment of the levator ani muscle imaging with 3D pelvic floor ultrasound, comparing it to MR imaging as the reference standard. Several studies using MR imaging pelvic floor ultrasound have demonstrated that levator defects occur after vaginal birth [10,12,15,17].

Because accurate assessment of pelvic floor injury may help explain symptoms and potentially guide future treatment options, it is important to study on imaging in detecting these levator defects.

Moreover using 3D pelvic ultrasound for diagnosing levator defects is non-invasive, patient friendly, less expensive and it has practical advantages like shorter examination time and less exclusion criteria like prosthesis, claustrophobia etc compared to MR-imaging.

Translabial 3D-ultrasonography allows imaging of the levator ani muscle including assessment of its integrity [11,20]. However until now no accuracy studies have been published concerning the use of 3D ultrasonography to diagnose levator defects. Few publications mention inter-observer variability in diagnosing levator defects. Steensma and co-workers have conducted a test-retest series of 50 volume datasets of patients with pelvic floor dysfunction for diagnosing levator defects to assess inter-observer reliability, which yielded a Cohen's kappa of 0.83 (95% Confidence interval (CI) 0.59,1.0), meaning excellent agreement [11]. In another study on grading the size of levator defects, the same research group has reported moderate agreement between different observers reflected in an intraclass correlation coefficient (ICC) of 0.53 (95% CI 0.13, 0.76) [20].

The main objective of our study is to estimate the diagnostic accuracy of translabial 3D ultrasonography of the pelvic floor as compared to MR imaging, the reference standard, for diagnosing levator defects in women with POP.

The second aim of this study is to estimate the level of agreement between observers and determine whether levator defects are a risk factor for recurrence after POP surgery. In addition, the cost-effectiveness of introducing translabial ultrasonography in the work-up of a patient with POP will be estimated in a decision analytic model.

## Methods/design

### Study aims

1. Estimating the diagnostic accuracy of translabial 3D ultrasonography of the pelvic floor as compared to MR imaging for diagnosing levator defects in women with POP.

2. Measuring the inter-observer agreement in diagnosing and in grading levator defects with 3D ultrasonography.

3. Measuring the association between levator defects and recurrent POP after anterior repair.

4. Evaluating potential cost-effectiveness of introducing 3D ultrasonography for diagnosing levator defects in the work-up of patients with POP.

### Study design

The TRUDIL study is a multicentre cohort study in nine teaching and non-teaching hospitals in The Netherlands.

### Ethical considerations

The study has been approved by the institutional review board of the Maastricht University Medical Centre, in Maastricht. Ethical approval for this study was obtained on 22-02-2010, number 08-2-093. Local approval was obtained in all participating centers.

### Identifying eligible patients

Eligible patients will be identified by gynecologists in each of the nine participating hospitals in The Netherlands.

**Figure 1:**
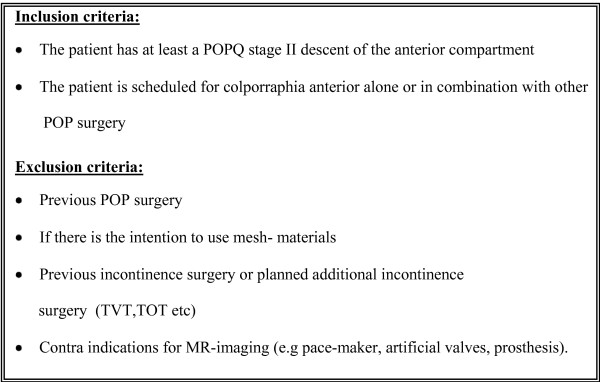
**Study inclusion and exclusion criteria**.

All consecutive women with at least a cystocele POPQ stage II who are scheduled for conventional anterior colporrhaphy alone or in combination with other POP surgery, without the use of mesh-materials, will be asked to participate in this study. Patients will be excluded in case of previous POP or incontinence surgery, in case of planned surgery with mesh materials, or in case of POP surgery in combination with incontinence surgery. Women with a contra-indication for undergoing MR imaging will also be excluded [see Figure 1].

All eligible patients will receive an information sheet about this study from their attending gynecologist. About one week after counseling has taken place, a research nurse or the gynecologist will call the patient to check for any upcoming questions about the study and her willingness to participate. If the patient is willing to participate, informed consent will be signed. The patient and the attending gynecologist will not be informed on the findings in the study until the study has been finished, therefore these findings do not affect the choice of treatment for these women.

### Interventions

#### Diagnostic work-up

Of each patient, baseline characteristics will be recorded. Participants will be interviewed and examined for prolapse by their attending gynecologist. POP will be staged according to the POPQ staging system of the International Continence Society [21].

All patients will undergo MR imaging and 3D ultrasonography before surgery takes place. The assessment of 3D ultrasound and MR-imaging of the levator ani muscle will be performed by experienced observers who have performed and assessed more than hundred 3D translabial ultrasounds or pelvic floor MR images before the start of the study.

All images will be stored according to a detailed protocol [see Additional files 1 and 2].

3D-ultrasound will be performed after emptying the bladder. 3D ultrasonographic volume data sets will be collected and stored. Afterwards, all volumes will be interpreted independently offline by two examiners who are blinded to the associated clinical data. If disagreement exists, an expert panel, which consist of three trained gynecologists, will decide on a final conclusion in a consensus meeting.

For reading the MR-images the same protocol will be followed. Images will be interpreted by two independent examiners and if disagreement exists consensus will be reached by three trained radiologists in a consensus meeting. The radiologists and the gynecologist are also blinded for each others assessment.

### Follow-up

Case record forms on the surgical data and complications will be completed. Follow-up appointments will be made six weeks, six months and twelve months postoperatively. At these measurement time points a physical examination for staging POP will be performed by an independent examiner to diagnose any anatomical recurrence. Validated Quality of Life and subjective feelings of recurrence questionnaires will be handed out before the surgery takes place and at six and twelve months after the surgery (EuroQol-5D, patient global impression of severity/improvement). For all these visits case record forms will be completed.

### Outcome measures

#### Primary outcome

The diagnostic test performance of translabial 3D ultrasonography as compared to MR imaging in detecting levator defects.

#### Secondary outcomes

1. Measuring the inter-observer agreement in diagnosing and in grading levator defects with 3D ultrasonography.

2. Measuring the association between levator defects and recurrent POP after anterior repair.

3. Evaluating potential cost-effectiveness of introducing 3D ultrasonography for diagnosing levator defects in the work-up of patients with POP.

### Data collection

The data will be recorded in a web based registry. Participants receive a case number to treat their data anonymously and to blind the observers. The observers are also blinded to all ultrasonography, MR-imaging data and clinical data. The patient and the attending gynecologist will not be informed of the findings in the study until the study has been finished.

### Sample size consideration

Ultrasound studies describe a prevalence of 20-40% of levator defects in patients with POP [15,17]. One MRI study detected levator defects in 55% of the women with POP. Calculating a sample size for the diagnostic test characteristics is complicated by the fact that only a few studies have been published about reproducibility of 3D ultrasound and in this study no other test characteristics were given. Assuming a sensitivity and specificity of 90% of 3D ultrasound for diagnosing levator defects in a population of 120 women with POP, with a prevalence of levator ani defects of 40%, we will be able to estimate predictive values with an accuracy of 10% below or above the point estimate of positive predictive values and negative predictive values.

Including 120 patients yields at least 80% to demonstrate that inter-observer agreement is substantial (Kappa >= 0.61), using a 0.05 significance level in one-sided testing, while expecting a 40% prevalence of levator defects and anticipating a value of the kappa statistic of 0.78 or better. In order to be able to detect a difference in recurrence rate after anterior colporrhaphy of 25% or more (35% in women with a levator defect vs 10% in women without levator defect), with an alpha of 5% and a power of 80%, a population of 102 patients with at least POPQ stage II prolapse in the anterior compartment, of whom 41 (40%) women with a levator defect, will be sufficient.

We are aiming to include 140 patients in this study, taking into consideration 3% technical problems in performing 3D-ultrasonography or MR imaging, which can result in non-evaluable images, and a further safety margin of 10%.

### Economic evaluation

Potential cost-effectiveness of translabial 3D ultrasound in the diagnostic work-up of a patient with POP to diagnose levator defects in order to identify patients at risk for recurrence, and subsequent adjustments of the type of surgery, will be determined by comparing diagnostic strategies. The incremental costs-effectiveness ratio(s) will be expressed as the incremental costs per recurrence avoided. As only part of the data needed to estimate potential cost-effectiveness of the 3D ultrasound will be collected empirically, a decision analytic model will be constructed [22,23]. The comparative sensitivity, specificity and costs of 3D ultrasound versus MR imaging for the diagnostic work-up of patients with POP will explicitly be incorporated in the model. The cost analysis will be performed from a hospital perspective, according to Dutch guidelines and will be estimated from study entry to twelve months follow up [24].

### Statistical analysis

Accuracy of 3D ultrasonography for diagnosing levator defects will be expressed in terms of sensitivity, specificity, standardized predictive values and their 95% -confidence interval. For the calculation of 95%-confidence intervals the Wilson formula for proportions will be used. ROC analysis will be performed for the grading of the size of levator defects with 3D ultrasound. Inter-observer and intra-observer agreement of diagnosing levator defects with 3D ultrasound will be evaluated by means of Cohen's Kappa coefficient and intra class coefficient. Logistic regression will be performed to determine whether levator defects constitute an independent risk factor for recurrence after POP surgery.

### Time plan

Patient recruitment started in March 2010 and is planned to continue until June 2011. The follow-up period is 12 months and therefore will continue until June 2012.

This study is conducted in cooperation with several centers collaborating in the Dutch Urogynecology Research Consortium. Most participating hospitals have a research nurse, who contributes to the counseling of the patient and the web-based data collection.

### Knowledge transfer

The outcome of this study will be important for the debate on the value of 3D ultrasound of the pelvic floor in a patient with pelvic organ prolapse for detecting levator defects. If the diagnostic accuracy of 3D ultrasonography in diagnosing levator defects and the clinical relevance of levator defects in this study will be confirmed, further implementation into guidelines and training of urogynecologists and ultrasonographers will be taken care of. It is also important to inform gynecologists if this relevance cannot be confirmed, if only to prevent any treatment based on a technique that is not relevant or accurate enough. The results of this study will be presented at national and international scientific meetings, and will be published in international scientific journals.

## Competing interests

The authors declare that they have no competing interests.

## Authors' contributions

MW, KK and JPR were responsible for the identification and formulation of the research question, and contributed to the development of the study protocol and study design.

All authors discussed the study design and commented on the protocol.

KN was responsible for the drafting of this paper. All authors have read and approved the final version of the manuscript.

## Pre-publication history

The pre-publication history for this paper can be accessed here:

http://www.biomedcentral.com/1472-6874/11/23/prepub

## Supplementary Material

Additional file 1 **Protocol Ultrasound**. Click here for file

Additional file 2 **Protocol MR imaging**. Click here for file
