# Supplementary material for: Protocol for Translabial 3D-Ultrasonography for diagnosing levator defects (TRUDIL): a multicentre cohort study for estimating the diagnostic accuracy of translabial 3D-ultrasonography of the pelvic floor as compared to MR imaging
Source: BMC Womens Health. 2011 Jun 3;11:23. doi: 10.1186/1472-6874-11-23 (PMC3121676; doi:10.1186/1472-6874-11-23)
Supplement: Additional file 1 — Protocol Ultrasound. [file 1472-6874-11-23-S1.DOC]

**Protocol Ultrasound:**

All patients will undergo a 3D ultrasound on a Voluson GE Kretz Ultrasound machine before surgery takes place. All ultrasound images will be made according to a strict protocol.

The ultrasound data sets will be stored anonymously.

The data sets are assessed by two independent examiners using 4D-view, an application of GE Kretz Voluson for offline assessments of ultrasound volume data sets. Both observers are blinded for all clinical data.

All datasets with discongruent judgments of the degree of trauma of the levator ani muscle, will be discussed in a consensus meeting joined by at least three experts in 3D/4D translabial ultrasound. The attending gynaecologist nor the patient is informed on the results of the ultrasound imaging before the follow-up of 1 year is completed.

**Settings GE Voluson ultrasound machine:**

In the main 2D setting the ultrasound recording is performed using an angle of 70˚, 2 focal zones and normal optimized tissue imaging (OTI normal). High speckle reduction imaging (SRI 4-6) is combined with high harmonic imaging (HI high). Images are further optimized by using Cross Beam Compound Resolution Imaging (X-Beam CRI 2-4). Converting into main 3D setting it is necessary to enlarge the angle as much as possible. On the GE Kretz Voluson 730 this will be 85˚, on the Voluson E8 this will be 90˚. The setting for quality has to be high.

**Protocol for performing 3D/4D pelvic floor ultrasound:**

Ultrasonography images are acquired using an 8-4 MHz transabdominal transducer covered with a latex condom (Voluson 730 expert, GE Kretz Ultrasound, Zipf, Austria). The translabial ultrasound examinations are performed with the patient in the supine position with slightly flexed legs. Patients will be requested to empty their bladder prior to the examination. No opacification of structures will be performed.

The transabdominal transducer is placed against the introitus. The transducer is manipulated until a precise midsagittal view is acquired in the 2D setting. The midsagittal orthogonal plane includes four reference points: the end of the central axis of the symphysis pubis, the urethra, the anorectal canal and the levator muscle. The ultrasound machine is then switched to the 3D setting and the box of interest is selected at the minimal anteroposterior diameter of the levator hiatus, from the posterior margin of the symphysis pubis to the anterior margin of the puborectal muscle where it defines the anorectal angle. Two cineloops of contractions of the pelvic floor are recorded and afterwards two cineloops of a valsalva manoeuvre are recorded. The beginnings of all four cineloops are showing the situation at rest.

**Assessment of the volume images:**

The recorded volumes will be analysed off-line using Kretz 4D-view V 5.1 software.

In a 4D-cine loop of a contraction of the pelvic floor the rendered images in the midsagital (A), frontal (B) and transversal (C) ortogonal plane are analysed. In the cineloop in the A-plane the moment of maximal contracted muscle is selected for analysis. The maximal contracted state is at maximal cranioventral displacement of the levator muscle in the midsagittal view. The box of interest in the midsagittal orthogonal plane includes the four reference points: the end of the central axis of the symphysis pubis, the urethra, the anorectal canal and the levator muscle. In the B-plane the symphysis pubis has to be seen symmetrically on both sides. In the C-plane the symphysis arc, the anorectal canal and the levator sling has to be visualised symmetrically on both sides.

If it is not possible to obtain these images, there possibly might exist a levator defect. Other contraction images must be analysed to exclude artifacts because of the recording process.

After acquiring the correct C-plane Tomographic Ultrasound imaging is performed. Tomographic Ultrasound Imaging (TUI) is a technique to show the levator ani muscle in multiple slices of 2.5 mm. With this technique levator damages can be assessed.

The levator muscle will be assessed in three different slices (reference image *, and the two more cranially slices +1 and +2) for the left and the right muscles separately. The slices are magnified by a factor 1.3.

The scoring is performed conform the method as described by Delancey for MR imaging. The percentage of missing muscle is estimated. If no damage is visible a score of 0 will be assigned, if less than half of the muscle is missing a score of 1 will be assigned, if more than half of the muscle is missing a score of 2 will be assigned, and if the complete muscle bulk is lost a score of 3 will be given. Then the scores are summed for the left and right side separately. A total score of 0 denotes no defect; a defect score of 1-3 is a minor defect except when there is a unilateral score of 3 indicating a major defect, score 4-6 indicates a major levator defect too.

Asymmetry which can not be resolved by manipulating the axes is suggestive for a unilateral defect. Lateralization of the vagina (like a hanging mouth or butterfly configuration) is another sign for levator defects. Thinning of the attachment of the levator sling to the symphysis pubis with normal configuration indicates a partial defect.

The urethral levator gap is assessed by measuring the distance between the center of the urethra and the most lateral parts of the attachment of the levator muscle to the ramus inferior of the symphysis pubis. Measurements in the reference and the two more cranially located slices (image*, +1 and +2) can reinforce a suspicion of a levator defect.

Finally hiatal area will be measured during rest, during contraction and at maximal valsalva.
