# Supplementary material for: Protocol for Translabial 3D-Ultrasonography for diagnosing levator defects (TRUDIL): a multicentre cohort study for estimating the diagnostic accuracy of translabial 3D-ultrasonography of the pelvic floor as compared to MR imaging
Source: BMC Womens Health. 2011 Jun 3;11:23. doi: 10.1186/1472-6874-11-23 (PMC3121676; doi:10.1186/1472-6874-11-23)
Supplement: Additional file 2 — Protocol MR imaging. [file 1472-6874-11-23-S2.DOCX]

**Protocol MR imaging**

The MR images will be made according to a strict protocol and images will be stored anonymously. All MR data are evaluated offline by two independent examiners according to protocol using a dicom viewer. The observers are blinded for all clinical data. If there are differences in judgment of the degree of trauma of the levator ani muscle, then a consensus meeting will be convened. Five trained radiologist will come to a final conclusion in this meeting.

The attending gynaecologist nor the patient is informed on the results of the ultrasound imaging before the follow-up of 1 year is completed.

**Protocol for MR scanning**

The MR imaging examination is performed with the patient in supine position with parallel and slightly flexed legs. Patients were requested to void for 0.5 h prior to their examination. No premedication is given. The urethra, bladder, vagina, and rectum are not opacified. MR images are acquired using either a 1.5 or 3 tesla MR scanner (Siemens/GE/Philips) and a surface coil. MR images of the pelvis are obtained in the sagittal plane using either a Half-Fourier acquisition single-shot turbo spin-echo sequence (2000 ms/90 ms repetition time/echo time; 150° flip angle) or true fast imaging with steady state precession. The entire pelvis from symphysis to second or third lumbar verbetrae is scanned Consequently axial and coronal T2-weighted turbo/fast spin echo sequence (>3500 ms, < 100ms repetition time/ echo time; 150° flip angle, slice thickness 3 mm and 0.5 x 0.5 in plane resolution)is acquired covering the entire pelvis from symphysis to the fifth lumbar verbetrae. In addition a 3-dimensional T2-weighted turbo/fast spin echo sequence (< 1 x 1 x 1 mm) may be acquired in addition.

**Assessment of the MR images:**

By using a dicom viewer all Mr images are assessed off line. In the axial and coronal plane the attachment of the levator muscle to the ramus inferios of the sympysis pubis is identified. Asymmetry which can not be resolved by manipulating the axes is suggestive for a unilateral defect. Lateralization of the vagina (like a hanging mouth or butterfly configuration) is another sign for levator defects. Thinning of the attachment of the levator sling to the symphysis pubis with normal configuration indicates a partial defect.

The scoring is performed conform the method as described by Delancey for MR imaging.(ref) The percentage of missing muscle is estimated. If no damage is visible a score of 0 will be assigned, if less than half of the muscle is missing a score of 1 will be assigned, if more than half of the muscle is missing a score of 2 will be assigned, and if the complete muscle bulk is lost a score of 3 will be given. Then the scores are summed for the left and right side separately. A total score of 0 denotes no defect; a defect score of 1-3 is a minor defect except when there is a unilateral score of 3 indicating a major defect, score 4-6 indicates a major levator defect too.

The urethral levator gap is assessed by measuring the distance between the center of the urethra and the most lateral parts of the attachment of the levator muscle to the ramus inferior of the symphysis pubis.

Finally hiatal area will be measured during rest, during contraction and at maximal valsalva.
